# Chitosan and Its Structural Modifications for siRNA Delivery

**DOI:** 10.34172/apb.2023.030

**Published:** 2022-01-08

**Authors:** Mona Y. Al-Absi, Anna Eleonora Caprifico, Gianpiero Calabrese

**Affiliations:** School of Life Sciences, Pharmacy and Chemistry, Kingston University London, Penrhyn Road, Kingston upon Thames, KT1 2EE, United Kingdom.

**Keywords:** Chitosan, Chitosan derivatives, siRNA, Nanoparticles, Gene therapy, Tumour

## Abstract

The use of RNA interference mechanism and small interfering RNA (siRNA) in cancer gene therapy is a very promising approach. However, the success of gene silencing is underpinned by the efficient delivery of intact siRNA into the targeted cell. Nowadays, chitosan is one of the most widely studied non-viral vectors for siRNA delivery, since it is a biodegradable, biocompatible and positively charged polymer able to bind to the negatively charged siRNA forming nanoparticles (NPs) that will act as siRNA delivery system. However, chitosan shows several limitations such as low transfection efficiency and low solubility at physiological pH. Therefore, a variety of chemical and non-chemical structural modifications of chitosan were investigated in the attempt to develop a chitosan derivative showing the features of an ideal siRNA carrier. In this review, the most recently proposed chemical modifications of chitosan are outlined. The type of modification, chemical structure, physicochemical properties, siRNA binding affinity and complexation efficiency of the modified chitosan are discussed. Moreover, the resulting NPs characteristics, cellular uptake, serum stability, cytotoxicity and gene transfection efficiency *in vitro* and/or *in vivo* are described and compared to the unmodified chitosan. Finally, a critical analysis of a selection of modifications is included, highlighting the most promising ones for this purpose in the future.

## Introduction

**Figure 1:**
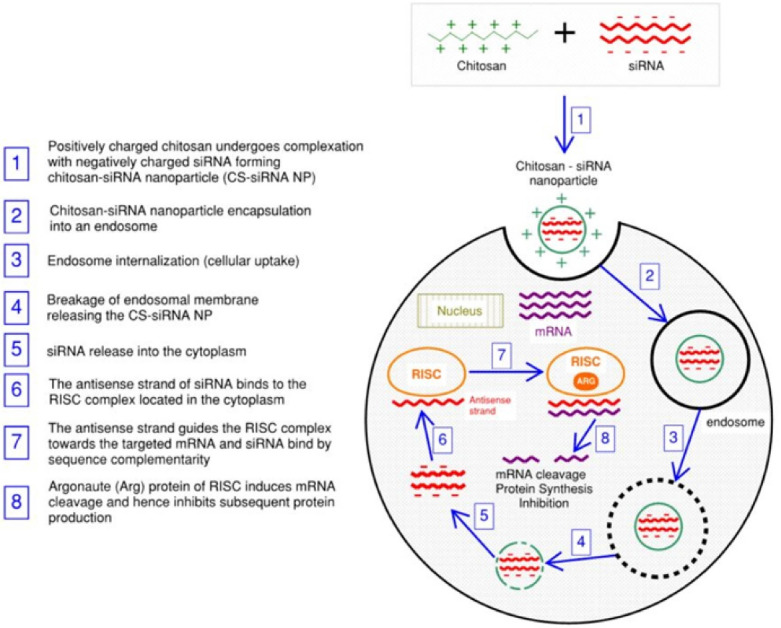
RNA interference mechanism explained using siRNA and chitosan nanoparticles.

 The genetic factor of cancer plays a key role in its development with more than 200 genes associated with its etiology.^1^ Therefore, gene therapy, in the form of gene silencing, has been implemented as a promising strategy for its treatment.^2^ Gene silencing employs RNA interference (RNAi) mechanism and has received particular attention in the last decade since, by interfering with the post-transcription phase of protein synthesis, it inhibits the production of mutated proteins underlying the genesis of cancer.^3^ Within the RNAi approaches, small interfering RNA (siRNA) is widely used due to the specificity of its mechanism. siRNA molecules are usually double stranded with 20-23 nucleotides in length.^4^ One of the siRNA strands, the antisense strand, guides a multiprotein complex called RNA interfering silencing complex (RISC) found in the cytoplasm, towards the targeted messenger RNA (mRNA) and mediate their binding by sequence complementarity mechanism. This targeted mRNA, which has a genetic sequence encoding the mutated protein, will undergo cleavage and degradation induced by a component of RISC, called argonaute 2 protein. This prevents the next stages of protein synthesis to occur hence inhibiting the expression of the disease-inducing protein and preventing disease progression (Figure 1).^5,6^

 In this process, the delivery of siRNA into the cell’s cytoplasm is a key step and different strategies have been adopted to deliver siRNA using carriers that allow efficient, safe, and repeated administration.^7,8^ Gene carriers can be either of viral or non-viral origins. Viral vectors have been the most frequently studied and used in clinical trials due to their higher gene transfection efficiency and levels of gene expression. However, the rising concerns about their safety made the non-viral vectors more suitable for the purpose.^9^

**Figure 2:**
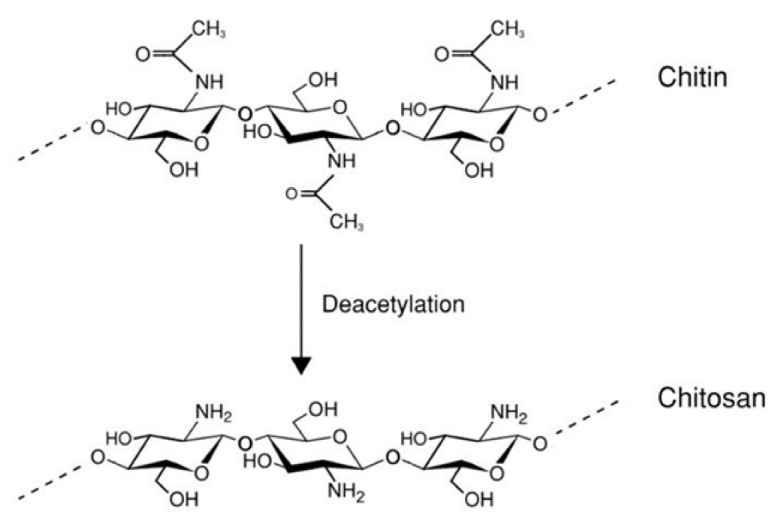
Chemical structure of chitosan obtained via deacetylation of chitin.

 Chitosan is a cationic polysaccharide of natural origin that is composed of β-(1-4)-linked-D-glucosamine and N-acetyl-D-glucosamine. It can be obtained by depolymerization and deacetylation of chitin (Figure 2), a polymer abundant in crustacean exoskeleton and fungi cell walls.^10^ Previous research identified chitosan as one of the most desirable polymeric carriers for siRNA, generating much interest in the recent years as a non-viral vector for gene therapy.^11^

 Chitosan has already shown its potential for siRNA delivery both in *in vitro* and *in vivo *investigations,^12^ and has already been regarded as the main constituent for siRNA nanocarriers.^7^ This is due to its favorable characteristics including biodegradability, biocompatibility, low immunogenicity, highly positive charge, high nuclease resistance, low cost, and susceptibility to structural modification.^2^ Some tumor features can be exploited to develop smart nanocarriers. For example, the mild acidic pH of the tumor encouraged the development of pH-sensitive chitosan nanoparticles (NPs) where siRNA will be released in a pH-dependent manner.^13^ Moreover, chitosan exhibits a mechanism called the “proton sponge effect” that allows siRNA endosomal escape: upon cellular encapsulation, chitosan/siRNA NPs increase the endosomal environment acidity, and the excess cationic charges provoke influx of water and chloride ions into the endosomes, neutralizing the positive charges. This results in an excessive osmotic swelling leading to the physical rupture of the endosomes, releasing the NPs in the cytosol. The proton sponge effect is responsible not only for the siRNA release from endosomes into the cytosol but also for inhibiting its premature lysosomal degradation.^8^

 Three most common types of NPs-based delivery systems include polymer-based, biomimetic-based and inorganic based nanocarriers.^14^ Chitosan is commonly used in the design of biomimetic NPs able to escape the immune system, prolonging their survival in the blood circulation.^14^ One of the most popular drug-loading methods used during NP formation, is non-covalent adsorption which includes different interactions such as ion-ion interactions, hydrogen bonding, van der Waals interactions, or hydrophilic and hydrophobic properties.^15^

**Figure 3:**
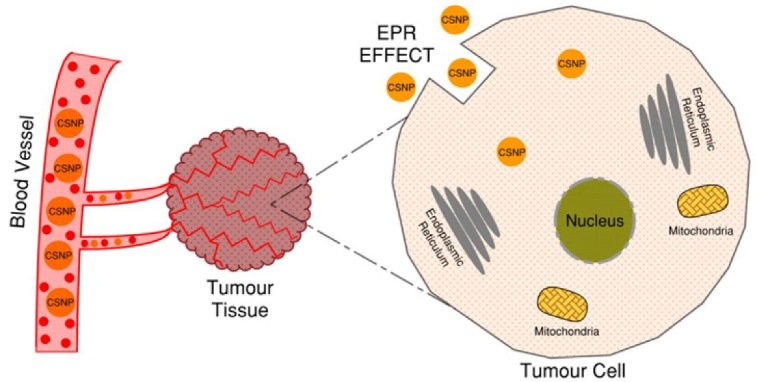
Enhanced permeation retention effect applied to chitosan/siRNA nanoparticles in the tumour tissue.

 The morphology (size and shape) and surface charge of the NPs influence their physicochemical properties, in turn affecting drug delivery efficiency.^14^ NPs with a size lower than 20 nm are removed by renal clearance whereas those with a size higher than 200 nm are recognized by the immune system.^16^ The ideal NP’s size able to pass through the leaky, fenestrated vasculature of the tumor vessels, is in the range of 50-100 nm.^15^ Since tumour’s lymphatic drainage is defective and inefficient, it allows NP’s retention in the interstitial fluids, stimulating the “enhanced permeation and retention effect” (EPR), described in Figure 3. The EPR is responsible for the selective accumulation of molecules of a given size (including NPs) in the tumour tissue rather than in the normal tissue, hence nanomedicines can rely on it to increase its therapeutic efficiency with minimal side effects.^16,17^

 Nevertheless, chitosan has its own drawbacks such as limited solubility since it is only soluble at pH < 6.5 at which the amino groups become protonated and able to form hydrophilic interactions with water. Therefore, at physiologic pH, the number of chitosan’s positively charged groups available for siRNA complexation is largely reduced. This impedes siRNA condensation into NPs and prevents particle stability *in vivo*. Moreover, chitosan’s transfection efficiency (namely the ability to deliver the desired gene or nucleic acid of interest into a specific cell) is low due to several factors. These include the tendency to aggregate and interact with biologic components such as serum proteins, resulting in decreased bioavailability, poor intracellular delivery and increased cytotoxicity.^8,18^

 To overcome these drawbacks and meet the requirements of an ideal siRNA carrier, chitosan’s structure has been studied thoroughly and possible chemical functionalizations were investigated to improve its physical and chemical characteristics. This review summarizes the structural modifications of chitosan that have been proposed within the last 10 years and have shown efficacy upon *in vitro* and/or *in vivo* testing in the context of cancer gene therapy. Moreover, the discussed modifications are compared, critically evaluated and the most prosperous ones highlighted for further research.

## Physicochemical properties of chitosan

###  Molecular weight

 Chitosan’s molecular weight (MW) affects NP’s size and stability in turn affecting their cellular uptake, siRNA release, and gene transfection efficiency. Chitosan with MW less than 10 kDa cannot form stable NPs whereas higher MW (25-50 kDa) results in more stable NPs.However, chitosan with higher MW yields larger NPs, leading to more inter-particle interactions and resulting in aggregation or colloidal instability.^19^ Conversely, low MW chitosan (LMWC) is characterized by a higher binding affinity and siRNA condensation capacity compared to high MW chitosan (HMWC). Consequently, upon complexation with siRNA, LMWC forms NPs with smaller hydrodynamic radius and a more regular, spherical shape.^19^ Moreover, LMWC/siRNA nanocomplexes showed enhanced cellular uptake and gene silencing efficiency than HMWC/siRNA nanocomplexes.^19,20^

###  Degree of deacetylation

 Chitosan’s degree of deacetylation (DDA) determines its positive charge and solubility. The DDA indicates how many free amino groups are available for interaction with siRNA affecting the cellular uptake and transfection efficiency of the resulting NPs. Chitosan with high DDA showed a positive effect on gene knockdown since more positive charges were available and the siRNA complexation efficiency increased.^9^ Alameh et al^21^ reported that nanocomplexes based on chitosan with low to intermediate DDA (72%, and 80%) had lower knockdown efficiencies (≈ 5%, 25%) than higher DDA (92% and 98%) resulting in enhanced efficiency (up to 80%).^21^

###  Polymer/siRNA molar ratio

 The polymer/siRNA molar ratio (N/P ratio) is defined as the number of nitrogen atoms in chitosan per phosphate atom in a gene hence the ratio at which chitosan binds to siRNA. This molar stoichiometry of chitosan-siRNA interaction determines the NP’s surface charge, in turn affecting stability, cell interaction and transfection efficiency.^9^ A high N/P ratio induces a greater proportion of chitosan to be available for complexation with siRNA resulting in a high complexation efficiency.^9^ Contrastingly, at low N/P ratio, the NP’s zeta potential decreases to neutral or negative values, leading to particle aggregation and inefficient cellular internalization and transfection.^9,21^

## Chemical modifications of chitosan

 Chemical modifications of chitosan might result in different transfection efficiencies according to the hydrophilic or hydrophobic nature of the chemical group attached: hydrophobic moieties tend to enhance complex formation, siRNA protection, and cell uptake; while hydrophilic moieties increase the solubility, reducing the cytotoxicity of the nanocarrier.^22^

###  Hydrophobic modifications

 Different alkyl chitosan derivatives were complexed with siRNA to form NPs. Longer side chains and much higher substitution degree showed a higher level of transfection, but a lower gene silencing efficiency. This was probably due to a greater amount of siRNA on the outer shell, which made NPs more exposed to serum nucleases.^8^ Another modification of chitosan involved the addition of secondary and tertiary amines, such as diethylaminoethyl (DEAE) group, to strengthen the interactions with siRNA, improving its condensation capacity and enhancing transfection efficiencies.^19^ Different DEAE content and DDA of chitosan were used to achieve more stable NPs with higher cellular uptake and cell viability.^23^ Furthermore, methylating the primary amino groups on chitosan’s structure improved the transfection efficiency.^24,25^ This was done by introducing a quaternary ammonium group into chitosan’s structure resulting in quaternized chitosan or by the quaternization of its amino groups resulting in *N, N, N*-trimethyl chitosan (TMC). These chemical modifications introduced secondary and quaternary amino groups to chitosan, increasing its solubility over a wider range of pH values, hence forming more stable polyplexes with increased siRNA’s transfection efficiency.^24-26^

###  Hydrophilic modifications

 Addition of polyethylene glycol (PEG) molecules to the surface of chitosan improved its solubility at physiological pH. Moreover, it enhanced nanocomplexes stability in both* in vitro* and *in vivo* studies, by shielding its positive charge, preventing protein corona formation and subsequent aggregation. As a result, the polymer’s bioavailability and half-life is increased by escaping the immune system activation with a reduction in polymer’s cytotoxicity.^27^ Moreover, PEG can also be used as a linker between the targeting moiety and the polyplex surface.^27^ However, it was found that the cellular uptake and gene transfection efficiencies of the PEG-chitosan/siRNA nanocomplexes were affected by the degree of PEGylation on chitosan’s backbone (PEG graft density/degree of substitution).^27,28^ As the value of degree of substitution increased, the gene knockdown efficiency decreased.^27^

## Functionalisation of chitosan with peptides

 Part of chitosan’s low transfection efficiency is due to its low intracellular delivery (poor ability to penetrate cells).^18^ To overcome this, cell-penetrating peptides (CPPs) were conjugated to chitosan. CPPs are short cationic or amphipathic peptides characterized by an intrinsic ability to enhance the cellular uptake of genes/proteins. CPPs bind covalently to chitosan’s structure enhancing its ability to adhere and penetrate cell membranes, improving the transfection efficiency of the resulting CPP-modified chitosan/siRNA complexes.^24^ Many CPPs (such as nona-arginine, protamine, poly-L-arginine, histidine, TAT- trans activated transcription factor, CGKRK pentapeptide) are currently being investigated for their cell-penetrating capabilities. Some of them are chemically synthesized (such as nona-arginine) while others have natural origins (such as trans activated transcription, TAT).

###  Synthetic cell penetrating peptides

 Synthetic CPPs are characterized by a high amount in arginine molecules, conferring strong positive charges that enhance the cell penetrating efficiency.^29^ For instance, the conjugation of chitosan with nona-arginine was shown to enhance the stability of the nanocarrier along with improving its cellular association and gene silencing efficiency.^30^ These effects were enhanced by adding a “spacer arm” made of glycine units between chitosan and nonarginine.^12^ Moreover, the conjugation of chitosan with poly-L-arginine resulted in an increased RNA delivery efficiency and was found advantageous given the intrinsic biodegradability and biocompatibility properties of poly-L-arginine.^31^ Patil et al^32^ generated stable NPs by conjugating chitosan to protamine which showed several advantages including enhanced membrane penetrating ability, protection of the RNA against nuclease and high binding affinity to RNA. Sun et al^29^ conjugated chitosan to histidine which showed high buffering capacity, increasing the proton-sponge effect.

###  Natural cell penetrating peptides

 Within CPPs of natural origins, TAT was found to have efficient cell penetrating features. TAT is the transcription activating factor of the human immunodeficiency virus (HIV), containing a domain responsible for its cell-penetrating properties, enhancing cellular uptake.^33^ Indeed, this domain is rich in arginine and lysine amino acid residues able to interact with the negatively charged cell surface proteoglycans, leading to a strong cell adherence, regardless of temperature, receptors, and energy-driven pathways.^26,33^ TAT may be attached covalently or non-covalently to cationic polymers such as chitosan. However, due to the steric hindrance occurring between the two cationic moieties (chitosan and TAT), a PEG molecule was used as a linker, protecting siRNA from degradation and stabilizing the resulting NPs.^28^ Yang et al^34^ employed glycol chitosan, showing higher targetability and gene silencing efficiency than PEGylated chitosan.

 A tumor-targeting cell penetrating pentapeptide group known as CGKRK was also conjugated to chitosan oligosaccharides. CGKRK showed high specificity towards angiogenic blood vessels and tumor cells. However, CGKRK alone did not interact readily with siRNA and hence it was hypothesized that hydrophobically-modified CGKRK improved siRNA’s tumor cell permeation and delivery.^6^ Different fatty acyl derivatives of CGKRK (Fa – CGKRK) were synthesized and subsequently proved for their ability to deliver siRNA inside the tumor.^6^ Some of these fatty acyl derivatives of CGKRK were complexed to chitosan, to enhance its transfection ability.^6,35^ Many of these fatty acyl CGKRK derivatives were proven to be efficient in siRNA binding, siRNA protection from degradative nucleases, selective targeting of breast and prostate cancer cell lines. The conjugates were evaluated for their silencing efficiency of a model protein involved in cancer cell proliferation (Kinesin spindle protein, KSP). Among all the fatty acid-CGKRK conjugates studied, Oleic acid-CGKRK conjugate showed the most significant knockdown of KSP (≈ 55-60%) resulting in efficient suppression of subcutaneous melanomas and ovarian tumors.^6^

## Addition of polyethyleneimine

 Polyethyleneimine (PEI) is a synthetic cationic polymer characterized by a strong positive charge and a repeating unit consisting of two ethylene spacers and one amino group.^36^ It has been widely used for nucleic acid delivery, and even though PEI-based nanocarriers showed higher transfection efficiency, it has been regarded as less safe than chitosan.^36,37^ Therefore, it was conjugated to chitosan yielding a polyplex with enhanced transfection efficiency while maintaining the cytotoxicity level to a minimum.^36^ Both *in vitro* and *in vivo* investigations showed an enhanced targeted gene silencing efficiency of chitosan-PEI based NPs.

## Discussion

Table 1 summarizes a selection chemical modifications of chitosan explored in this review, regarding transfection efficiency, serum stability, *in vitro* and *in vivo* gene silencing.

**Table 1:** Summary of the explored chemical modifications of chitosan in the context of small interfering RNA delivery

**Moieties added to chitosan**	**Transfection efficiency**	**Serum stability**	***In vitro*** ** gene silencing**	***In vivo*** ** gene silencing**	**Ref.**
Alkyl groups	67-75%	Partial degradation after 48 h	30%	N/A	^8^
Diethylaminoethyl	Effective	Enhanced	80-90%	N/A	^19^
Quaternized	N/A	N/A	70%	N/A	^24^
Trymethyl groups	85-90%	100%	40-70%	N/A	^25^
Poly-L-arginine	Effective	Enhanced	80%	Effective	^31^
TAT-glycol	Effective	N/A	70%	Effective	^34^
CGKRK	75%	91-98%	55-60%	N/A	^6,35^
PEI	Effective	Enhanced	80%	Effective	^36^

**Figure 4:**
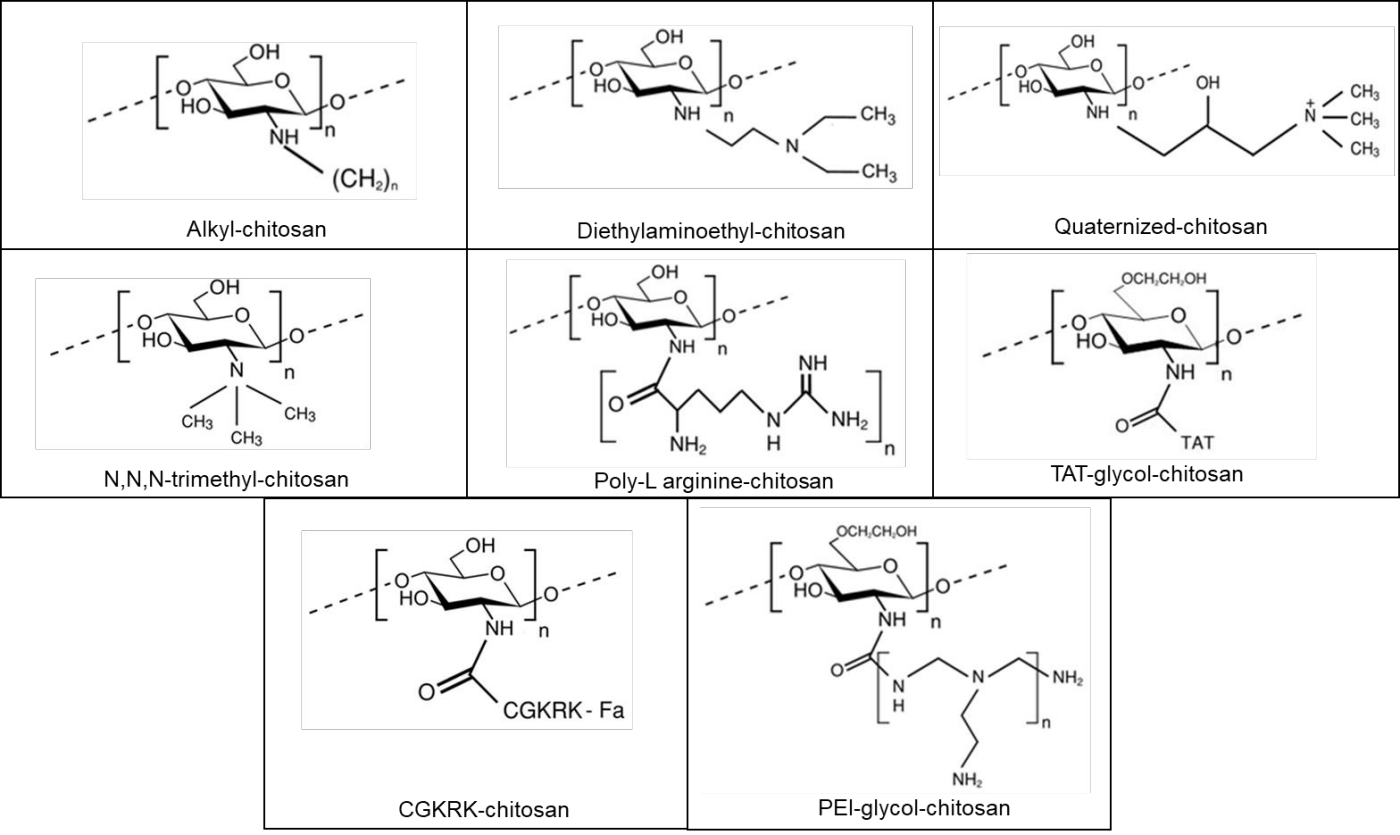
Chemical structures of chitosan derivatives in the context of small interfering RNA delivery.

 The structures of the chemical modifications of chitosan explored are shown in Figure 4. These modifications yielded chitosan derivatives, which upon complexation with siRNA, resulted in an enhancement of chitosan/siRNA NP’s physicochemical and gene transfection related properties (such as cellular uptake, siRNA release, or knockdown efficiency). Despite analogous principles of characterisation, a comparison of the described modifications may be hampered by several parameters affecting experimental conditions (such as pH and presence of serum), different cell lines used for the *in vitro* investigations, and variable molecular properties of chitosan.

 Upon complexation with siRNA, chitosan derivatives generated NPs having a size within the acceptable range for tumour cell penetration (10-500 nm),^15^ except for nona-arginine/chitosan and CGKRK-chitosan, whose NP’s size (within 600 nm and 800 nm, respectively) fallen outside the acceptable range.^30,35^

 Regarding zeta potential, all chitosan derivatives showed positive zeta potentials enabling them to bind to the negatively charged siRNA. However, protamine-chitosan had the lowest value of + 4 mV^32^ and alkyl-modified chitosan showed the highest value (up to + 38 mV).^7^ NPs exhibited spherical morphology except CGKRK-chitosan derivative, whose particles were fibrous and network-like in shape.^31^ Regarding the cellular uptake, it was enhanced in most of the resulting NPs, especially those based on TMC showed the highest cellular uptake (up to 90%). Furthermore, the most stable NPs in the presence of serum proteins were those based on CGKRK-chitosan, resulting in the highest percentages (91.6–98%) of intact siRNA retained.^35^ Regarding cytotoxicity, values differed upon variation of siRNA concentration, for example, a siRNA concentration of 250 μg/mL resulted in 100% viability, while at higher concentration (1 mg/mL), cell viability decreased up to 70%.^32^

 The* in vitro* transfection efficiencies was also evaluated in the modified chitosan-based NPs, showing enhanced gene silencing with varying degrees: especially, alkyl-, diethylaminoethyl-, PEG-, nona-arginine-, and PEI-derivatives resulted in gene-silencing percentages equal to or higher than 75%.^8,18,27,30,36^ However, not all the investigated chitosan derivatives were assessed for *in vivo* gene silencing. The protamine group was evaluated based on the rat’s lung tissue weight increase and bronchial epithelium degeneration.^33^ The PLR-group and PEI-group were evaluated for their RFP protein expression reduction,^31,36^ while TAT- and poly(histidine-arginine)_6_ groups were evaluated on tumour-bearing mice, where slower tumour growth was observed.^29,34^ These groups are suitable candidates to undergo further *in vivo* studies and subsequent clinical trials. However, some of them would need further characterization studies due to the semi-satisfactory results obtained. For example, poly(histidine-arginine)_6_-chitosan/siRNA NPs, TAT-PEG-chitosan/siRNA and TAT-glycol chitosan NPs were not tested for their serum stability,^29^ while PLR-chitosan/siRNA NPs size was not within the ideal range for easy tumour penetration.^25^ From the studies explored, protamine-chitosan/siRNA and PEI-glycol chitosan/siRNA NPs may be the best candidates for future *in vivo* studies and subsequent clinical trials.

## Conclusion

 Chemical functionalization of chitosan showed very promising results as a non-viral gene vector, suggesting the forthcoming success of a novel anti-cancer approach. An overview of the most recent chemical modifications of chitosan describing their implication and role in gene silencing was provided and the afore-mentioned chemical modifications of chitosan have not been clinically approved yet. Nevertheless, there are some chitosan derivates that have already undergone *in vivo* studies and are candidates for upcoming clinical trial examination.

## Competing Interests

 The authors declare no conflict of interest.

## Ethical Approval

 There are no ethical issues

## References

[1] Seyhan AA (2011). RNAi: a potential new class of therapeutic for human genetic disease. Hum Genet.

[2] Chuan D, Jin T, Fan R, Zhou L, Guo G (2019). Chitosan for gene delivery: methods for improvement and applications. Adv Colloid Interface Sci.

[3] Chery J (2016). RNA therapeutics: RNAi and antisense mechanisms and clinical applications. Postdoc J.

[4] Mahmoudpour M, Ding S, Lyu Z, Ebrahimi G, Du D, Ezzati Nazhad Dolatabadi J (2021). Aptamer functionalized nanomaterials for biomedical applications: recent advances and new horizons. Nano Today.

[5] Schiffelers RM, Mastrobattista E. Oligonucleotides. In: Crommelin DJ, Sindelar RD, Meibohm B, eds. Pharmaceutical Biotechnology: Fundamentals and Applications. 3rd ed. New York: Informa Healthcare USA, Inc; 2008. p. 211-24. 10.3109/9781420044386.

[6] Sharma M, El-Sayed NS, Do H, Parang K, Tiwari RK, Aliabadi HM (2017). Tumor-targeted delivery of siRNA using fatty acyl-CGKRK peptide conjugates. Sci Rep.

[7] Lee SJ, Huh MS, Lee SY, Min S, Lee S, Koo H (2012). Tumor-homing poly-siRNA/glycol chitosan self-cross-linked nanoparticles for systemic siRNA delivery in cancer treatment. Angew Chem Int Ed Engl.

[8] Villar-Alvarez E, Leal BH, Martinez-Gonzalez R, Pardo A, Al-Qadi S, Juarez J (2019). SiRNA silencing by chemically modified biopolymeric nanovectors. ACS Omega.

[9] Cao Y, Tan YF, Wong YS, Liew MWJ, Venkatraman S (2019). Recent advances in chitosan-based carriers for gene delivery. Mar Drugs.

[10] Mao S, Sun W, Kissel T (2010). Chitosan-based formulations for delivery of DNA and siRNA. Adv Drug Deliv Rev.

[11] Fernandes JC, Qiu X, Winnik FM, Benderdour M, Zhang X, Dai K (2012). Low molecular weight chitosan conjugated with folate for siRNA delivery in vitro: optimization studies. Int J Nanomedicine.

[12] Jeong EJ, Choi M, Lee J, Rhim T, Lee KY (2015). The spacer arm length in cell-penetrating peptides influences chitosan/siRNA nanoparticle delivery for pulmonary inflammation treatment. Nanoscale.

[13] Ashrafizadeh M, Delfi M, Hashemi F, Zabolian A, Saleki H, Bagherian M (2021). Biomedical application of chitosan-based nanoscale delivery systems: potential usefulness in siRNA delivery for cancer therapy. CarbohydrPolym.

[14] Ding S, Khan AI, Cai X, Song Y, Lyu Z, Du D (2020). Overcoming blood-brain barrier transport: advances in nanoparticle-based drug delivery strategies. Mater Today (Kidlington).

[15] Ding S, Zhang N, Lyu Z, Zhu W, Chang YC, Hu X (2021). Protein-based nanomaterials and nanosystems for biomedical applications: a review. Mater Today.

[16] Ku SH, Kim K, Choi K, Kim SH, Kwon IC (2014). Tumor-targeting multifunctional nanoparticles for siRNA delivery: recent advances in cancer therapy. Adv Healthc Mater.

[17] Narmani A, Jafari SM (2021). Chitosan-based nanodelivery systems for cancer therapy: recent advances. CarbohydrPolym.

[18] de Souza R, Picola IPD, Shi Q, Petrônio MS, Benderdour M, Fernandes JC (2018). Diethylaminoethyl- chitosan as an efficient carrier for siRNA delivery: improving the condensation process and the nanoparticles properties. Int J Biol Macromol.

[19] Holzerny P, Ajdini B, Heusermann W, Bruno K, Schuleit M, Meinel L (2012). Biophysical properties of chitosan/siRNA polyplexes: profiling the polymer/siRNA interactions and bioactivity. J Control Release.

[20] Alameh M, Dejesus D, Jean M, Darras V, Thibault M, Lavertu M (2012). Low molecular weight chitosan nanoparticulate system at low N:P ratio for nontoxic polynucleotide delivery. Int J Nanomedicine.

[21] Alameh M, Lavertu M, Tran-Khanh N, Chang CY, Lesage F, Bail M (2018). siRNA delivery with chitosan: influence of chitosan molecular weight, degree of deacetylation, and amine to phosphate ratio on in vitro silencing efficiency, hemocompatibility, biodistribution, and in vivo efficacy. Biomacromolecules.

[22] Choi C, Nam JP, Nah JW (2016). Application of chitosan and chitosan derivatives as biomaterials. J Ind Eng Chem.

[23] Martins GO, Segalla Petrônio M, Furuyama Lima AM, Martinez Junior AM, de Oliveira Tiera VA, de Freitas Calmon M (2019). Amphipathic chitosans improve the physicochemical properties of siRNA-chitosan nanoparticles at physiological conditions. CarbohydrPolym.

[24] Faizuloev E, Marova A, Nikonova A, Volkova I, Gorshkova M, Izumrudov V (2012). Water-soluble N-[(2-hydroxy-3-trimethylammonium)propyl]chitosan chloride as a nucleic acids vector for cell transfection. CarbohydrPolym.

[25] Liu X, Ma L, Qin W, Gao C (2013). Effect of N/P ratios on physicochemical stability, cellular association, and gene silencing efficiency for trimethyl chitosan/small interfering RNA complexes. J Bioact Compat Polym.

[26] Mohammadi Z, Eini M, Rastegari A, Tehrani MR (2021). Chitosan as a machine for biomolecule delivery: a review. CarbohydrPolym.

[27] Guţoaia A, Schuster L, Margutti S, Laufer S, Schlosshauer B, Krastev R (2016). Fine-tuned PEGylation of chitosan to maintain optimal siRNA-nanoplex bioactivity. CarbohydrPolym.

[28] Yang C, Gao S, Dagnæs-Hansen F, Jakobsen M, Kjems J (2017). Impact of PEG chain length on the physical properties and bioactivity of PEGylated chitosan/siRNA nanoparticles in vitro and in vivo. ACS Appl Mater Interfaces.

[29] Sun P, Huang W, Kang L, Jin M, Fan B, Jin H (2017). siRNA-loaded poly(histidine-arginine)(6)-modified chitosan nanoparticle with enhanced cell-penetrating and endosomal escape capacities for suppressing breast tumor metastasis. Int J Nanomedicine.

[30] Park S, Jeong EJ, Lee J, Rhim T, Lee SK, Lee KY (2013). Preparation and characterization of nonaarginine-modified chitosan nanoparticles for siRNA delivery. CarbohydrPolym.

[31] Noh SM, Park MO, Shim G, Han SE, Lee HY, Huh JH (2010). Pegylated poly-l-arginine derivatives of chitosan for effective delivery of siRNA. J Control Release.

[32] Patil S, Bhatt P, Lalani R, Amrutiya J, Vhora I, Kolte A (2016). Low molecular weight chitosan–protamine conjugate for siRNA delivery with enhanced stability and transfection efficiency. RSC Adv.

[33] Malhotra M, Tomaro-Duchesneau C, Saha S, Kahouli I, Prakash S (2013). Development and characterization of chitosan-PEG-TAT nanoparticles for the intracellular delivery of siRNA. Int J Nanomedicine.

[34] Yang F, Huang W, Li Y, Liu S, Jin M, Wang Y (2013). Anti-tumor effects in mice induced by survivin-targeted siRNA delivered through polysaccharide nanoparticles. Biomaterials.

[35] El-Sayed NS, Sharma M, Aliabadi HM, El-Meligy MG, El-Zaity AK, Nageib ZA (2018). Synthesis, characterization, and in vitro cytotoxicity of fatty acyl-CGKRK-chitosan oligosaccharides conjugates for siRNA delivery. Int J Biol Macromol.

[36] Huh MS, Lee SY, Park S, Lee S, Chung H, Lee S (2010). Tumor-homing glycol chitosan/polyethylenimine nanoparticles for the systemic delivery of siRNA in tumor-bearing mice. J Control Release.

[37] Molinaro R, Wolfram J, Federico C, Cilurzo F, Di Marzio L, Ventura CA (2013). Polyethylenimine and chitosan carriers for the delivery of RNA interference effectors. Expert Opin Drug Deliv.

